# Exploring Loneliness among Korean Adults: A Concept Mapping Approach

**DOI:** 10.3390/bs14060492

**Published:** 2024-06-11

**Authors:** Soo-Jung An, Young-Seok Seo

**Affiliations:** 1 Department of Psychotherapy, Myongji University, Seoul 03674, Republic of Korea; tiaresoo@mju.ac.kr; 2 Faculty of Education, Yonsei University, Seoul 03722, Republic of Korea

**Keywords:** loneliness, Korean adults, concept mapping, conceptual elements

## Abstract

In South Korea, the proportion of adults experiencing severe loneliness has been increasing rapidly. Accordingly, this study examines the elements of loneliness experienced by Korean adults and investigates their structural relevance using concept mapping. Korean adults (47) were recruited for individual in-depth interviews based on their scores on the UCLA Loneliness Scale. The interviews yielded 80 unique statements, which were then evaluated using multidimensional scaling and a hierarchical cluster analysis. A cluster map of loneliness was derived, with three clusters: (1) emotional distress due to the actual or anticipated absence of connection in relationships, (2) emotional distance from oneself or from others in a relationship, and (3) powerlessness and emptiness due to being directionless. Two dimensions distinguished these clusters: the lack of a sense of connection or self-assurance, and an inward or outward focus. These findings reveal that loneliness encompasses more than unmet relational needs; it also involves self-attentional focus, indicating a need to reconceptualize the notion of loneliness. The study’s implications extend to counseling theory and practices by highlighting the importance of addressing both relational connections and self-perceptions in interventions for loneliness. By expanding the understanding of loneliness through empirical data, this research provides a more comprehensive framework for addressing loneliness.

## 1. Introduction

Modern psychologists describe loneliness as a “21st century epidemic”, recognizing it as a significant clinical issue [1]. In the UK, approximately 9 million people suffer from loneliness [2], leading to an estimated annual cost of $25 billion for healthcare services and other related factors [3]. In South Korea, 4 in 10 adults experience severe loneliness, with this proportion rapidly increasing each year [4,5,6]. One study reported that 77% of Koreans feel lonely [7]. Despite living in an era of hyper-connectivity, individuals today still experience a sense of disconnection. 

Loneliness can lead to dissatisfaction with life [8], hinder the experience of happiness [9], and result in psychological and emotional maladaptation, including depression, anxiety, helplessness, and anger [10,11]. Loneliness is also associated with behavioral problems such as addiction, eating disorders, and escapism [12,13], and can be a factor in suicidal impulses and actions [14]. Furthermore, loneliness can impact physical health, including stress pathways, blood sugar levels, obesity, and immune function, thereby accelerating aging [15,16]. The threat of loneliness extends beyond individual issues and is a significant societal problem.

What is loneliness and what are its elements? Despite the exponential growth in emotional research over the past decades, loneliness remains one of the least conceptually agreed-upon topics [17,18]. Exploratory, qualitative research is, thus, required to systematically and integratively define it [19,20,21]. In particular, a discussion on the dimensions of loneliness is crucial for understanding its conceptualization. The widely used University of California Los Angeles (UCLA) Loneliness Scale was developed based on the assumption that loneliness is a culturally universal phenomenon differing only in frequency and intensity [22]. Though this scale has the advantage of clearly and fundamentally revealing the essence of loneliness, there has been criticism of the fact that it is difficult to measure the dimensions and types of loneliness experienced in various relational contexts by focusing solely on its quantity and intensity [19,23,24,25]. Other scales (e.g., the Social Emotional Loneliness Scale for Adults; [25]), based on Weiss’s dual classification system [26], categorize loneliness into emotional loneliness and social loneliness, depending on whether there is a deprivation of intimacy and attachment or a lack of solidarity and affiliation in social relationships [23,27]. 

In addition, some argue for a three-dimensional understanding by adding physical loneliness [28], suggesting that, despite feeling emotionally and socially connected, the inability to be in the physical presence of others when needed can lead to loneliness. A discussion on existential loneliness, reflecting the fundamental alienation that humans face from birth to death, has also increased [21,29]. However, research confirming the dimensions of loneliness from an integrative perspective has been lacking since Weiss’s dual classification [26], and discussions on these dimensions are fragmented. 

Seo et al. aimed to distinguish loneliness from similar concepts such as isolation [30], solitude, exclusion, and relationship dissatisfaction along two dimensions: “choice” (active–passive) and “perception” (subjective–objective). They found that loneliness was relatively clearly differentiated from objective states such as isolation and voluntary solitude. Moreover, loneliness is identified as having distinct and structural characteristics that separate it from other emotions and phenomena [31]. However, the distinction between loneliness and similar concepts remains ambiguous. Some scholars differentiate between “objective isolation”, representing the absence of social activity and disconnection from social networks, and “subjective isolation”, that is, an individual’s perceived lack of social support [32,33]. According to these classifications, distinguishing loneliness, conceptualized as emotional pain due to perceived relational deficiency, from subjectively perceived isolation is not straightforward. This implies the need for more detailed discussions on the essential concepts and unique elements to clarify loneliness.

Furthermore, considering Korea’s sociocultural context, the loneliness experienced by Koreans may have different characteristics from that experienced by Westerners. Following Benedict and Herskovits’ advocacy for cultural relativism, the proposition that individual emotions should be understood in various cultural contexts has been widely accepted [34]. Within the context of their interactions with their environment, individuals ascribe personal meaning to their relational situation, leading to distinct emotional experiences based on that meaning [35,36]. According to Vuong [37], the Mind sponge Theory posits that individuals absorb and filter information based on their core values, which are shaped by their cultural background. Accordingly, Koreans experience loneliness within the sociocultural context of Korea, despite it being a universal emotion. This provides a framework for understanding how core sociocultural values influence the cognitive processes that lead to mental products such as perceived loneliness. For example, in East Asian nations, where humility is a core cultural value, individuals tend to filter out overly positive self-expressions, maintaining a more reserved presentation of emotions, including loneliness, compared to Western nations [38]. This highlights the possibility of cultural differences in the structure and context of emotional experiences. 

The impact of sociocultural context on the experience of loneliness is evident in both cross-cultural differences and historical contexts [39]. Loneliness, which was perceived as homesickness or isolation in the 16th century, became a social psychological concept focusing on interaction with others by the 19th century. This indicates the need to reconstruct loneliness into a contemporary definition that reflects current contextual and temporal aspects [17].

Comparative studies have revealed that the way individuals experience loneliness can vary between individualistic and collectivistic cultural contexts [40]. For instance, loneliness appears to be most pronounced among young men in individualistic cultures, particularly in Western societies [41]. However, members of collectivistic cultures, who seek strong bonds within their in-group, experience more loneliness if these bonds are lacking [42,43]. The inconsistency in the results of cross-cultural comparisons on loneliness has been attributed to the lack of comprehensive conceptual agreement, as comparative studies have been conducted based on criteria from specific cultural contexts without achieving universal consensus [44]. Given the dominance of collectivistic culture among Koreans, it is necessary to explore the essential elements and dimensions of loneliness experienced by Koreans as a basis for comparing how loneliness is experienced in different cultures. 

Seo et al. conducted an analysis of previous studies [30], literary and philosophical works, newspaper and broadcast articles, and TV programs to examine how Koreans experience and represent loneliness. Inferring that there would be unique, intense types of loneliness experienced in Korea’s sociocultural context, they identified “collective loneliness”, “other-oriented loneliness”, and “fusional loneliness” as types of loneliness that Koreans prominently experience. Collective loneliness refers to the pain felt when individuals do not feel sufficiently embraced within meaningful groups, such as academic, geographical, or family bonds. In Korean society, where interpersonal relationships are highly valued, this type of loneliness is common. [30]. Other-oriented loneliness refers to the feelings of bitterness that arise when individuals compare their lives against those of others or societal standards rather than their own internal standards, leading to feelings of inadequacy and emptiness [30]. This goes beyond mere jealousy or social comparison. Instead, Koreans may experience a profound sense of isolation and diminished self-worth, feeling emotionally distanced and alone despite being surrounded by people. For instance, in a country characterized by the rapid development of the Internet and frequent overuse of social media, it has been shown that individuals who use social media extensively experience more intense loneliness than those who do not [30,45]. Finally, fusional loneliness occurs when individuals closely adhere to another person’s values and aspirations to the extent of losing their sense of self, leading to feelings of emptiness [30]. This is often seen in over-attached parent–child relationships, where relational attachment, rather than deficiency, induces loneliness. However, though Seo et al. revealed the culture-specific characteristics of loneliness among Koreans [30], they analyzed literature and secondary data and did not collect any data directly.

In summary, an inductive, exploratory study is necessary to understand how Koreans experience and perceive loneliness. Previous research and theories have not sufficiently explained the elements of loneliness, and empirical research conceptualizing loneliness in Korea’s sociocultural context is lacking. This study collected and analyzed empirical data on how Korean adults perceive loneliness to confirm the conceptual structure of loneliness experienced by Koreans. It also sought to identify common structural features found in previous research and reveal the unique elements of loneliness that are prominent among Korean adults.

Previous research has employed literature and theoretical analyses [19,31,46], primarily focusing on the researcher’s understanding to examine the components of loneliness. However, we used concept mapping to explore individuals’ experiences [47], clarify the abstract and subjective concept of loneliness, and, hence, lay a foundation for scholarly and practical interest in loneliness.

This study examined how Korean adults aged 20 and above perceived loneliness and explored the dimensions and components of loneliness based on participants’ language, highlighting the relevant and meaningful aspects of loneliness. Considering previous research that has observed differences in the experience of loneliness due to sociocultural context, we visually represented the differences in the perceived relevance of loneliness factors by gender, age, and occupation. The following research questions were explored: (1) What are the components of loneliness experienced by Korean adults? This question addresses the identification of loneliness components through the analysis of statements provided by the participants. (2) What are the dimensions that distinguish loneliness and what are the clusters that are derived from these dimensions? This question involves identifying the dimensions and clusters of loneliness using a conceptual map, which visually represents the various aspects of loneliness and the relationships between them. (3) What relative importance do Korean adults assign to the various loneliness elements (clusters and dimensions)? This question evaluates the relative importance of the identified clusters and dimensions, determining which elements are considered most significant by the participants. 

## 2. Materials and Methods

In this study, we employed the concept-mapping methodology [48], an exploratory and inductive approach, to comprehensively understand participants’ empirical perceptions of loneliness. Given the lack of a well-defined conceptual framework for loneliness, we deemed the concept-mapping method suitable for our research as it allows for a detailed depiction of conceptual structures. This methodology offers the advantage of mapping the empirical elements of participants’ experiences of loneliness and interpreting qualitative data though statistical techniques. It is particularly useful for addressing complex, abstract, and subjective phenomena like loneliness, which are difficult to express in simple and clear terms [49]. 

Following Kane and Trochim [48], we employed concept mapping to collect and analyze data. The procedure was as follows: (a) selecting participants and formulating the focus prompt through literature research on loneliness; (b) conducting in-depth interviews and extracting elements of loneliness by analyzing interview content; (c) structuring extracted statements by sorting and rating their similarities and relevance; (d) running two-dimensional multidimensional scaling (MDS) and hierarchical cluster analysis (HCA) to determine a final cluster solution; (e) labeling the clusters and interpreting the meaning of clustering; and (f) comparing the relevance between subsets of participants [48]. 

### 2.1. Preparation

#### 2.1.1. Participants

To generate statements on the loneliness experienced by Korean adults, we conducted individual in-depth interviews with 47 adult men and women aged 20 years or older in South Korea. Participants were recruited based on self-reported scores of 47 or above on the UCLA Loneliness Scale [50,51], indicating that they frequently or deeply experienced loneliness in their daily lives. The UCLA Loneliness Scale was developed without a validated cutoff point but was based on the relative comparison concept in which higher scores indicate a greater degree of loneliness. Previous studies utilizing the UCLA Loneliness Scale generally consider participants with total scores in the 80th percentile (top 20%) as being highly lonely. In studies with samples of adults aged 18 and above, college students, and older adults, the cutoff points for loneliness have ranged from 42 to 47 points [52,53,54]. In Korean studies, older adults experiencing moderate loneliness were classified as those who scored 35 or above on the UCLA Loneliness Scale [55], whereas college students scoring 41 or above were categorized as lonely [56]. Building on these studies, we adopted the most conservative criterion, setting the cutoff point at 47, and recruited participants accordingly.

As adults may experience loneliness differently depending on their social and environmental contexts, such as life course, marital status, and organizational affiliations [57,58], we recruited participants from diverse groups. We selected participants from various groups, including college students, full-time homemakers, full-time employees who had been receiving salaries from companies for at least one year, and older adults aged 65 and above, defined by the Korean government as the senior age group. This diverse pool of participants allowed us to collect ideas from different social contexts. According to Kane and Trochim [48], there is no strict requirement for the number of participants in concept-mapping studies, although 10 or more is appropriate. Similarly, Johnsen et al. recommended 10 to 20 participants [59]. Furthermore, a meta-analysis conducted by Choi [60] on previous studies using the concept-mapping method found that the number of participants in studies collecting qualitative interview data ranged from 11 to 20. Consequently, we aimed to recruit at least 10 participants, ensuring an even distribution across different age groups as much as possible.

The data were collected for two months from January to March 2022. During this time frame, we posted a recruitment flyer containing details about the study’s purpose, objectives, participation conditions, and methods on Internet forums with over 2 million members (e.g., Everytime, Momsholic, etc.). Those who wished to participate were asked to submit an online application form. Applicants were provided with a Google link containing the screening questionnaire (UCLA Loneliness Scale). Of the 92 applicants who submitted the application form, 7 (7.6%) did not complete the screening questionnaire, and 14 (16.5%) did not exceed the cutoff point in the screening results. Subsequently, we contacted applicants by phone to inform them of their eligibility for the interviews and provided additional information on the research purpose, expected duration, and participants’ rights. After obtaining their consent, the interview schedules and formats were co-ordinated, and some participants were excluded: seven (9.9%) did not meet the participation criteria (e.g., employed for less than one year), five (7.0%) misunderstood the research purpose (e.g., misunderstood as receiving a counseling service), two (2.8%) preferred only a phone interview instead of a video interview, and three (4.2%) were on psychotropic medication. We chose video interviews over phone interviews to capture non-verbal cues, build better rapport with participants, and verify the identity of participants, which was crucial for discussing their emotional experiences. Ultimately, data were collected from 47 individuals (51.1%). We obtained prior approval from the Institutional Review Board of the affiliated university and conducted the research in accordance with its research ethics standards (Approval No. 7001988-202203-HR-1504-03).

The participants’ average age was 40.49 years (range = 22–75 years). Among them, 15 participants (31.9%) were in their 20s, 10 (21.3%) were in their 30s, 11 (23.4%) were in their 40s, 3 (6.4%) were in their 50s, and 8 (17.0%) were 60 or above. There were 30 female participants (63.8%) and 17 male participants (36.2%). In total, 14 (29.8%) were college students, 13 (27.7%) were employed, 13 (27.7%) were full-time homemakers, and 7 (14.9%) were older adults. The participants had an average score of 56.70 (range = 47–76) on the UCLA Loneliness Scale. They mostly lived alone or with 1–4 cohabitants. There was no significant correlation between the number of cohabitants and the UCLA Loneliness Scale scores.

#### 2.1.2. Formulation of the Prompt

The focus prompt used in the in-depth interviews guided the brainstorming and development of statements on the concept of loneliness. The focus prompt was formulated based on a review of the literature on loneliness, loneliness-related scale items [21,49,61,62], and suggested concept-mapping procedures [48]. The final focus question for this study was: “What do you think loneliness is? Please freely share specific moments, situations, and emotions when you felt loneliness most intensely or frequently”. To confirm the question’s appropriateness, three counseling professors with experience in concept-mapping studies and assisting individuals who expressed loneliness were consulted. One consultant recommended providing an operational definition of loneliness, based on a literature review. However, loneliness is a universally recognized emotion that people intuitively understand [63]. Offering a predefined definition could potentially constrain participants, limiting their exploration of their subjective experiences and perceptions of loneliness. To avoid hindering the study’s purpose, which was to inductively explore how individuals experience loneliness, we decided not to provide an operational definition.

### 2.2. Statement Generation

#### 2.2.1. In-Depth Interviews

This step aimed to generate a large set of ideas from participants’ responses during the in-depth interviews in order to produce statements that represented the concept of loneliness [48]. Participants were asked to reflect on their experiences of loneliness based on the focus questionnaire, which they received a week before the interviews. At the beginning of the interviews, the researcher and participants verbally confirmed the research participation explanation and consent form, and both signed a copy, which was kept separately. Subsequently, using the focus questionnaire as a guide, participants recalled specific situations when they experienced loneliness and shared ideas related to the focus question. In concept-mapping research, brainstorming is encouraged to generate ideas in response to the focus question [48]. Therefore, we played a facilitating role in encouraging participants to produce various ideas related to loneliness, while striving to avoid limiting their thoughts and emotions, allowing them to express their experiences as they truly were. When participants had difficulty generating ideas, we provided supplementary questions and guiding statements to help them think about loneliness from different perspectives. We also summarized or reflected participants’ ideas back to them in the form of questions to ensure an accurate understanding of what they meant.

The interviews lasted approximately 50 to 90 min. The interview contents were recorded with participants’ consent, and personal information was represented using symbols or numbers. The audio from these recordings was transcribed verbatim and used for data analysis. When nuances of the participants’ reported text were unclear, the video recordings were reviewed to interpret non-verbal cues such as tone, facial expressions, or gestures, providing additional context. The recorded data were stored on a separate password-protected external hard drive. Interview were conducted until theoretical saturation was reached.

#### 2.2.2. Extracting Elements of the Concept

After conducting the in-depth interviews, we transcribed the interview content in its original language, Korean, and coded it in Excel. Only then were the final statements translated in to English by the researchers. Our aim was to maintain the participants’ precise words and meanings as much as possible. Depending on the participants, 21 to 49 basic statements were extracted. Following Kane and Trochim’s recommendation to avoid practical constraints in the process of integrating common ideas [48], we limited the number of basic statements to 100 or fewer. We applied the approach used by Bedi and included only statements that were mentioned by two or more participants in the list [47]. We also excluded statements that were deemed not directly related to loneliness or contained overly specific personal circumstances.

This process was used to supplement the existing list of statements with additional ideas regarding situations, cognition, and emotions related to loneliness and create a more comprehensive concept. This study defined emotions as activations that require important event stimuli, undergo cognitive evaluation, and comprise emotions, expressions, and bodily reactions [64]. Therefore, we determined that statements related to the emotional aspect of loneliness should include complex elements of situations, cognition, and emotions.

We independently reviewed the statements categorized according to key terms to ensure that they appropriately reflected loneliness, and then discussed them together. We also distinguished between statements reflecting the consequences of loneliness or the essential nature of loneliness. We strived to avoid biases or preconceptions about loneliness and to accurately reflect what the participants meant while reading the raw data. We did not exclude statements from multiple participants of experiences commonly associated with loneliness that aligned with existing definitions of loneliness. The final list of statements was reduced to 128.

To ensure the statements were clear and coherent, they were examined by a counseling professor, a holder of a doctorate in counseling, and a doctoral student in counseling. For instance, the statement “There is little interaction within the organization, and no one supports me”, was divided into two different ideas: “There is little interaction within the organization” and “There is no support within the organization”. Similar statements with similar meanings (e.g., “Feeling bitter when people close to me do not stand by me” and “Feeling like I am left alone when people close to me do not understand my feelings”) were combined into one statement (e.g., “Feeling like I have no one on my side if people close to me do not empathize with me”).

In total, 80 unique statements were identified. These statements were reviewed by four participants, one from each group. The participants were asked to provide feedback on whether they found that any statements were unclear or had overlapping meanings and whether any statements inadequately reflected loneliness. Apart from suggestions for minor modifications in some expressions, the participants generally agreed with the statements. 

### 2.3. Structuring of Statements

#### Sorting for Similarities and Rating for Relevance

Participants who had previously participated in the in-depth interviews were invited to participate in sorting the statements. Some participants were excluded due to difficulties with using computers or Excel, or personal circumstances (e.g., being diagnosed with COVID-19). Ultimately, 26 participants participated in this process. Additionally, six counseling experts (two women and four men) who had experience attending to clients with loneliness were involved in the sorting and rating tasks. The experts’ average age was 39.67 years (range = 35–46 years). All six experts were counseling professors or clinical psychologists working in university counseling centers, with an average counseling experience of 11.83 years.

A total of 32 individuals (participants and experts) took part in the similarity sorting and relevance rating process. The participants’ average age was 36.06 years (range = 22–75 years), with 11 in their 20s (34.4%), 9 in their 30s (28.1%), 8 in their 40s (25.0%), 2 in their 50s (6.3%), and 2 aged 60 or older (6.3%). Among them, 20 were women (62.5%), 12 were men (37.5%), 14 were employed (43.8%), 10 were university students (31.3%), 6 were full-time homemakers (18.8%), and 2 were senior citizens (6.3%). In total, 2 (6.3%) had completed high school, 10 (31.3%) were attending university, 6 (18.8%) held a Bachelor’s degree, 11 (34.4%) had a Master’s degree, and 3 (9.4%) had a doctoral degree.

**Figure A1:**
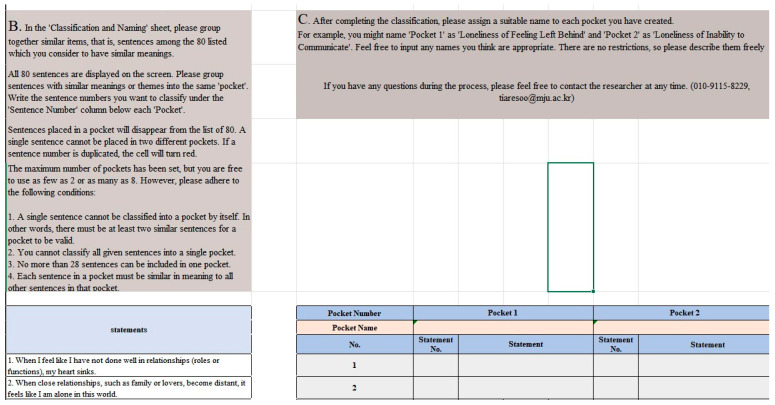
Excel sheet used by participants, which was in Korean, to sort the statements.

The participants received instructions and an Excel file for the statement sorting and rating tasks (please refer to Appendix A Figure A1 for additional information). Additionally, we provided guidance on the statement sorting and rating methods via telephone and encouraged participants to contact us if they encountered any difficulties or had questions during the tasks. We met with three participants who found it challenging to work online in person to conduct the sorting and rating tasks, while following COVID-19 prevention measures. We prepared 80 statement cards (measuring 21 cm × 4 cm) and presented them to the participants, explaining the sorting and rating methods.

Participants were first asked to sort the 80 statements in a way “that made sense to them” and group together statements that they “considered to be similar in meaning”. The sorting guidelines were as follows [48]: (a) each statement card could only be placed in one pile; (b) all cards must be placed in at least one pile; (c) participants could create as many piles as they wished, but each card should be grouped with at least one other card that had a similar idea about loneliness; and (d) there should be no leftover statements after the sorting process. We also applied Bedi’s rule that no more than 33% (27 cards) of the statement cards could be included in one single pile [47]. After grouping the cards, participants were asked to give a name to each pile that best represented its content. Participants grouped 9 statements on average, with the number of statements in each pile ranging from 4 to 20. Six participants classified the statements into six piles, which was the most common grouping.

After the statement sorting, participants were also asked to rate the relevance of each statement using a five-point Likert scale (1 = not relevant at all, 5 = very relevant) to assess “how relevant and meaningful each statement is perceived by Koreans”.

### 2.4. Concept-Mapping Analysis

This step involved analyzing the results of the sorting activity and generating maps by applying MDS and HCA [48]. 

#### 2.4.1. Multidimensional Scaling Analysis

We created individual similarity matrices sized 80 × 80 for each participant, corresponding to the number of participants. The 32 individual similarity matrices were then combined to create a group similarity matrix. Based on the group similarity matrix, MDS analysis was conducted using the Alternating Least Squares sCALing (ALSCAL) method. The ALSCAL method is one of the prominent techniques in MDS that places objects in a space using their relative distances, minimizing the discrepancy between the given distance matrix and the predicted distance matrix from the model to find the optimal positions for each object [65]. 

#### 2.4.2. Hierarchical Cluster Analysis

Following a two-stage clustering method [66], we conducted a clustering analysis using the x and y co-ordinates of each statement obtained through multidimensional scaling. We performed HCA using Ward’s algorithm to classify statements into clusters with similar concepts and determine the optimal number of clusters [48]. This method is particularly useful for interpreting data in concept-mapping studies, as it classifies clusters based on distance [48]. The cluster boundaries around groups of points mean that the statements are more frequently sorted together.

To ensure the validity of the determined number of clusters and confirm the appropriateness of clusters and sub-clusters, we then conducted additional cluster analyses using the average linkage and centroid linkage methods for hierarchical clustering, and the K-means method for non-hierarchical clustering. Comparing the results, the clusters’ appropriateness was confirmed as the statements in each cluster were highly identical. Descriptive statistical analyses were also conducted on the participants’ ratings of the final statements, and the results were presented by item, cluster, and group means for comparison.

To establish the reliability of this concept-mapping study, the split-half reliability was calculated following the methods proposed by Bedi and Trochim [47,67]. The participants were randomly divided into two groups based on their assigned numbers (odd and even), and the group similarity matrices (GSMs) of these two groups were used to calculate the split-half reliability. The correlation of GSM between the odd-numbered participants (*n* = 16) and even-numbered participants (*n* = 16) was 0.68 (*p* < 0.001), with a Spearman–Brown corrected value of 0.81. Moreover, the stress index of the two groups was compared, resulting in a stress index of 0.283 for the odd-numbered participants and 0.328 for the even-numbered participants. The appropriate stress index when using multidimensional scaling analysis is suggested to be between 0.205 and 0.365 [48]; the present study’s stress index met these criteria. Finally, based on the ratings of the statements, the average rating-to-reliability was calculated, and the Cronbach’s α was 0.922.

### 2.5. Interpretation of the Map

The relationships among the statements depicted in the concept map were interpreted through the above procedures. Meaningful names were given to statements positioned at the extremes of each dimension, and their respective meanings were interpreted. Additionally, the contents of statements within each cluster were reviewed, and clusters were named and interpreted based on the relevance of the statements, their relative positions, and the names provided by participants during the statement similarity sorting process. This process was further refined with the advice of one counseling professor. Finally, a group pattern matching approach was used to compare the average relevance across groups.

## 3. Results

### 3.1. Dimensions of Loneliness Experienced by Korean Adults

Based on the similarity matrices derived from 47 participants from Korea, an MDS analysis was performed to calculate the measure of agreement and explanatory power according to each dimension. Higher stress indices indicated a greater disagreement between the raw data rated by the participants and distances between the points, and that the maps did not accurately reflect the data. However, lower stress indices indicated a better validity with a higher measure of agreement [48,68]. In addition, a stress and squared correlation (RSQ) value, which is similar to the coefficient of determination in regression analysis, of ≥0.60 indicates a high explanatory power [69]. This study’s MDS analysis showed a two-dimensional stress index of 0.282 (*R*^2^ = 0.60), within the appropriate range for concept mapping.

Stress indices were represented as dots in a stress scree plot. Kruskal suggested selecting the dimension number at the first elbow of the stress plot, that is, the point at which the measure of agreement does not increase significantly even if the dimension number increases [70]. The stress index showed the most significant decrease at two dimensions, and the decrease in value leveled off from three dimensions and higher. Considering the interpretability, simplicity, and efficiency of expression, we determined that two dimensions were appropriate for this study. 

In a concept-mapping study, participant-generated statements are positioned on a two-dimensional plane based on their conceptual relatedness. The two dimensions represent underlying themes, and the clusters are formed by grouping statements that are close to each other on the map. We examined the overall statements distributed on both sides of the *X* and *Y* axes. The left side of the *X*-axis contained statements related to emotional experiences in relationships with others, specifically, where a sense of distance or unfulfilled connection was felt. In particular, it comprised statements explaining emotions experienced when desiring a sense of connectedness in intimate relationships but perceiving it as lacking. The right side of the *X*-axis contained statements related to loneliness experienced in social life, including missing out on important things due to a busy routine, anxiety about an uncertain future, feeling left behind or stagnant, and other emotions not related to specific objects. This direction comprised statements explaining emotions experienced when perceiving a lack of self-positivity or a lack of strong self-worth in social life. The first-dimension axis was, thus, related to individuals’ perceptions of “lacking” something in their relationships with others and society, and was named “Lack”; the two directions were named “Lack of Feeling Connected” and “Lack of Assurance”.

The negative direction of the *Y*-axis included statements that described emotional experiences of an absence of emotional intimacy and affection and the absence of someone to rely on or share important aspects of oneself with, thus representing loneliness that originates from the absence of emotional connection and sharing. Meanwhile, the positive direction of the *Y*-axis contained statements related to feeling that one’s desires or values are not sufficiently respected, representing loneliness originating from within oneself. The second dimension was, thus, named “Focus” since it highlighted the source of loneliness, whether from external (others) or internal factors (oneself). Hence, the directions were named “Outward” and “Inward” focus.

### 3.2. Clusters and Concept Map of Loneliness Experienced by Korean Adults

**Figure 1:**
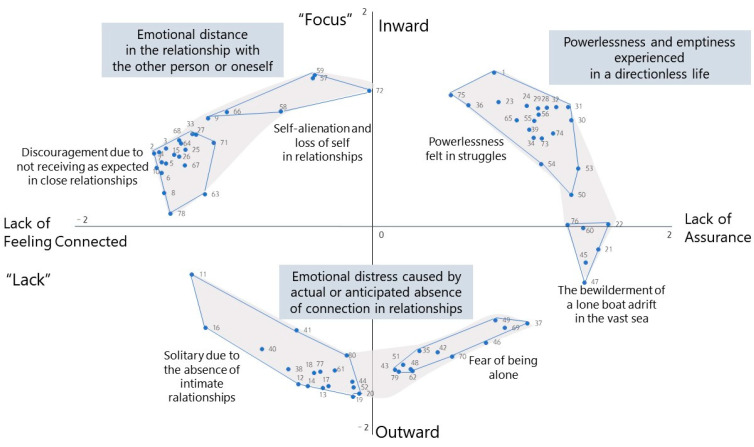
Concept map of loneliness experienced by Korean adults. Note: The numbered dots represent the statements derived from the study.

HCA (Ward’s method) was used to classify the statements about loneliness into clusters according to their relevance and similarity. A non-hierarchical clustering analysis (K-means method) was conducted, with the results showing that three clusters were appropriate. In addition, the results from the average linkage and centroid linkage methods showed that the clusters could be further divided into two sub-clusters each, considering the similarity of items within clusters and the conceptual clarity of the clusters. Ultimately, we derived a total of six sub-clusters in three clusters of loneliness experienced by Korean adults. The contents of statements in each cluster were reviewed to select a name for the cluster that precisely represented its contents. The criteria and names that participants assigned to the piles were also considered. Figure 1 shows the concept map, depicting the clusters in two dimensions.

Cluster 1 (“Emotional distress due to actual or anticipated absence of connection in relationships”), located across Quadrants 3 and 4, comprised 29 (36.3%) statements. This cluster consisted of feelings of solitude, emptiness, and fear of being alone without connection in relationships. Cluster 1 was divided into two sub-clusters. The first, “Solitude due to the absence of intimate relationships”, included statements related to emptiness and solitude due to the absence of a connection with others who have shared interests or a desire for support during difficult times. The second sub-cluster, “Fear of being alone”, included statements related to the fear of being alone or feeling lonely due to the lack of connection in meaningful relationships or groups that foster trust, support, and a sense of belonging. 

Cluster 2 (“Emotional distance from oneself or from others in a relationship”), located in Quadrant 2, comprised 26 (32.5%) statements. Of the sub-clusters in Cluster 2, “Dissatisfaction in a relationship due to unmet expectations” was closer to the *X*-axis (“Lack”). This sub-cluster included emotions of oppression, disappointment, and distance from others due to not being understood or accepted. The sub-cluster “Self-alienation and loss of self in relationships” was closer to the *Y*-axis (“Focus”). This sub-cluster included the experience of alienation from oneself due to loss of identity (e.g., one’s desires and hopes) while doing things for loved ones.

Finally, Cluster 3 (“Powerlessness and emptiness due to being directionless”), located across Quadrants 1 and 4, included 25 (31.3%) statements. This cluster comprised feelings of powerlessness and emptiness experienced when an individual feels that their existence or worth is not firmly rooted. The sub-cluster “Powerlessness felt during difficulties”, located in Quadrant 1, was closer to the *Y*-axis (“Inward”). This sub-cluster included statements related to a lack of self-assurance due to comparison with others, criticism from others, and lack of achievement. The sub-cluster “Bewilderment like a boat adrift in a vast sea” was closer to the *Y*-axis (“Lack of assurance”), which included anxiety from feelings of drifting alone in life. 

The statements, organized by cluster, and the mean and standard deviation of the relevance of each statement are provided in the Appendix A. Among the 80 statements, the participants rated the following as most relevant and meaningful: “There is no one I can deeply share my thoughts and feelings with” (*M* = 4.44), “I feel worthless when my worth is not acknowledged” (*M* = 4.38), and “I feel like I have to deal with my emptiness or difficult emotions on my own” (*M* = 4.28). Cluster 2 had the highest relevance (“Emotional distance from oneself or from others in a close relationship”, *M* = 3.74). The sub-clusters “Dissatisfaction in a close relationship due to unmet expectations” (sub-cluster in Cluster 2) and “Powerlessness felt during difficulties” (sub-cluster in Cluster 3) had the highest mean relevance. 

### 3.3. Pattern-Matching Analysis of Loneliness by Groups

Loneliness may be experienced differently depending on gender, socio-environmental context, and developmental stage within the life cycle [54,71]. Accordingly, we performed a pattern-matching analysis by sub-cluster to compare the mean relevance according to the participants’ characteristics.

**Figure 2:**
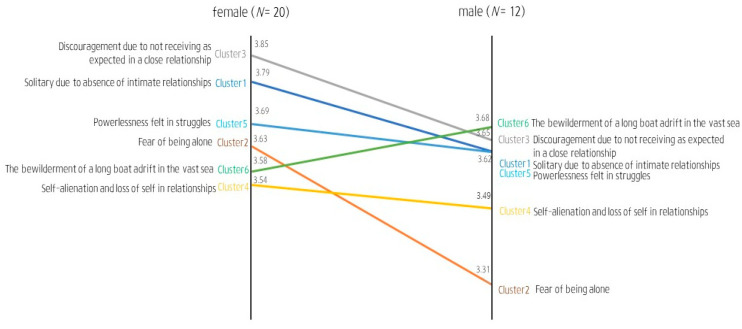
Pattern matching according to participants’ genders.

Comparing the mean relevance by gender (Figure 2), female and male participants perceived the sub-clusters “Dissatisfaction in a relationship due to unmet expectations” in Cluster 2 and “Bewilderment like a boat adrift in a vast sea” in Cluster 3 to be the most meaningful elements of loneliness, respectively. Women gave statistically significantly higher meaning to the sub-cluster “Fear of being alone” in Cluster 1 (*F* (1, 30) = 3.490, *p* < 0.05).

**Figure 3:**
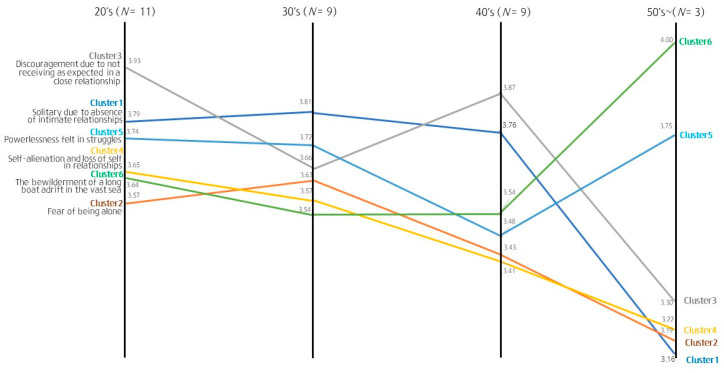
Pattern matching according to participants’ ages.

Comparing the mean relevance by age (Figure 3), participants aged 20–29 and 40–49 years perceived the sub-cluster “Dissatisfaction in a relationship due to unmet expectations” in Cluster 2 to be the most meaningful. Participants aged 30–39 years perceived “Solitude due to the absence of intimate relationships” to be the most relevant, whereas those aged ≥50 years rated this cluster as the least important. Participants aged ≥50 years perceived “Bewilderment like a boat adrift in a vast sea” to be the most relevant, whereas those aged 20–39 years perceived it to be the least relevant. The results also showed that there were statistically significant differences in the relevance of “Self-alienation and loss of self in relationships” (*F* (3, 28) = 3.715, *p* = 0.023), “Powerlessness felt during difficulties” (*F* (3, 28) = 3.854, *p* = 0.020), and “Bewilderment like a boat adrift in a vast sea” (*F* (3, 28) = 3.855, *p* = 0.020) according to age.

## 4. Discussion

This study used concept mapping to analyze 80 statements representative of the loneliness experienced by the Korean participants. Following similarity sorting and relevance rating, the statements were classified into two dimensions (“Lack” and “Focus”), three clusters (“Emotional distress due to actual or anticipated absence of connection in relationships”, “Emotional distance from oneself or from others in a relationship”, and “Powerlessness and emptiness due to being directionless”), and six sub-clusters (“Solitude due to the absence of intimate relationships”, “Fear of being alone”, “Self-alienation and loss of self in relationships”, “Powerlessness felt during difficulties”, and “Bewilderment like a boat adrift in a vast sea”).

### 4.1. Feeling of Lack Experienced by Koreans and Its Focus

We found that participants conceptualize loneliness based on a lack of feeling connected in relationships (“lack”) and whether their interest was focused inward or outward in relationships (“focus”). Participants experienced loneliness when they perceived that they had unmet relational needs or when they were not “assured” of their position within a social network. This finding was consistent with loneliness as defined by the social needs approach, which emphasizes relational deficiencies [26], and the cognitive perspective, which emphasizes subjective assessments and perceptions of situations [33]. The results support previous research indicating that loneliness should be conceptualized by integrating these approaches [30,31,72]. 

Previous studies have conceptualized “lack” as a passive state resulting from unfulfilled needs, specifically due to “not receiving” what is desired. However, in this study, “lack” is conceptualized as the regret of “not providing” help when someone close is lonely or facing difficulties. In other words, participants experience a sense of lack in various ways beyond the deprivation of needs or dissatisfaction with relational expectations [26,33], leading to loneliness. For instance, participants reported feeling lonely “when loved ones are going through a tough time and I can only watch because I cannot help them” (66) and “when loved ones feel lonely or hurt because of me” (68). These statements illustrate how their sense of “not providing” in relational contexts contributes to their feeling of loneliness. In other words, “lack” is interpreted as a state where one feels incomplete or unfulfilled internally, leading to a sense of emptiness and longing. When individuals do not receive what they desire, they experience this internal emptiness as “lack”. Similarly, when individuals are unable to provide help to someone they care about, they experience a sense of inadequacy and deficiency, which also contributes to feelings of “lack”. This inability to help loved ones leads to a perception of one’s own insufficiency and amplifies feelings of loneliness. Many Koreans exhibit a dominant subjective self, desiring to be central and influential in their relationships [73]. In this study, participants appeared to experience a sense of lack similar to a loss of faith in their own worth when they felt unable to demonstrate meaningful power in relationships and social networks. Desperation from feeling stagnant in comparison to others, emptiness from feeling alienated in an unfulfilling role, and bewilderment due to an unclear social standing caused the participants to feel they lacked something. Humans seek meaning or value to enrich their relationships or lives and draw the strength to accept suffering [74]. However, the perception of “failure” in not finding such meaning or value can lead to feelings of worthlessness. This perception was not a major topic in previous studies that have explained loneliness by focusing on relational needs and deficiencies, and isolation. However, these are important aspects in understanding loneliness among the Korean adult participants. 

This study also showed that the participants’ experiences of loneliness varied depending on whether their perspectives were inwardly or outwardly focused. In other words, the relationship with oneself and with others in living a precarious life was linked to loneliness. The “focus” dimension in this study demonstrated that loneliness is experienced within the context of an individual focusing on themselves or others, which is consistent with the premise that feelings are directed at a target [75]. Loneliness can, thus, be viewed as a targeted emotion focused on someone or something (i.e., either self-focused or other-focused). Other-focused refers to attention being directed toward the outside world. Having someone with whom to share feelings or to give mutual assistance and responses from others leave a strong impression, and loneliness is experienced from a negative interpretation of this. This conclusion is consistent with that of McGraw, that the relational context with others forms the basis for experiencing loneliness [76]. Conversely, self-focus is directed toward one’s own inner world and the interpretation of the self or relationship with the self, rather than with others. In this context, loneliness is experienced when an individual perceives a loss of self-worth due to their own or others’ negative perceptions of themselves. As such, self-worth, which enables individuals to perceive themselves as worthy or have a sense of identity, is linked to loneliness. Particularly in Korean culture, which emphasizes relational orientation, individuals my feel a deep loneliness and a sense of “failure” when they perceive that they are not making a significant impact on others or society and are not meeting their expectations. 

### 4.2. Loneliness Due to Relationship with Self, Others, and the World

We identified three clusters. The first, “Emotional distress due to actual or anticipated absence of connection in relationships”, reflected solitude, the feeling of being alone due to an absolute or a relative absence of others who can satisfy the fundamental relational needs for human connection (e.g., “There is no one I can deeply share my thoughts and feelings with; (12)”, and “It feels like I cannot belong or establish roots anywhere (46)”). This cluster included the most core characteristics of loneliness, such as “relationship”, “lack”, and “suffering”, consistent with previous research that has emphasized the structural characteristics of a lack of relational and social connection as the source of loneliness [77,78]. It is also consistent with McGraw’s claim that the “absence of others” cannot be ignored when conceptualizing loneliness [76], and with the analogy of loneliness as a social hunger or thirst [13]. In addition to relational lack and deficiency, a sense of isolation or fear of isolation from actual or symbolic social networks also amplifies such hunger. Being excluded or isolated from relationships or groups with which the values of life are shared is a core emotional element of loneliness for those who strive to form a community with others [79]. 

The second cluster “Emotional distance with oneself or with others in a relationship” reflected the feeling of a sense of distance and deficiency in a relationship despite the presence of someone close (e.g., “It is disappointing when the other person does not care about me as much as I care about them; (25)”, and “I feel bitter when I cannot express my own hardships due to a desire to support the other person (9)”). Similarly, Motta observed humans experiencing loneliness despite being in a close relationship [75], which intensifies loneliness due to unmet expectations of intimacy and empathy. This cluster is consistent with studies that highlight quality over quantity as a risk factor for intensifying loneliness [80,81].

Previous studies have categorized loneliness as emotional, social, and collective loneliness [26,82]. This study conceptualized loneliness based on the presence or absence of (qualitatively fulfilling) relationships. Relational needs, which include emotional intimacy, social interests, and groups for Koreans, overlapped to some extent. Some participants desired emotional connections with their coworkers beyond the social connection, and some older adult participants perceived social networks as relationships for sharing emotional intimacy. This indicates that Koreans tend to perceive both personal and social relationships as intimate psychological spaces, having greater emotional expectations from those who are close. In other words, the Korean adult participants perceive a relationship in terms of whether it is “close” or not, rather than by type, and have high expectations for acceptance, including “complete understanding”, “being on their side”, and “paying attention to them”, but “not being disappointed” or “having minor misunderstandings”. This exemplifies the Korean characteristic of forming a deep sense of solidarity and affection for people within the boundaries of “I” and “we” and exhibiting familial relational collectivism [83], including friendship, intimacy, and affection toward others [84]. In a relational context, Koreans who wish to exert their influence as central beings have high expectations from those close to them and desire to reaffirm closeness through the acceptance of such expectations [85]. Meanwhile, the likelihood of disappointment and despair from unmet expectations is also high. 

Furthermore, participants reported experiencing profound loneliness “when they felt they could not belong or put down roots somewhere” (46), and “when they felt they would remain alone without forming a family” (69), reflecting the collective identity of Koreans. In a collectivistic culture that emphasizes cohesion and solidarity, as seen in the reports of participants who felt they must receive unconditional support from close contacts or meaningful groups, individuals can more rapidly absorb loneliness when these expectations are unmet. 

Moreover, distance is created not only in relationships with others but also in the relationship with the self. The sense of distance from themselves that individuals feel when they experience alienation in a relationship and a loss of their own desire is consistent with loneliness in developing a sense of belonging, which is considered a culturally unique form of loneliness among Koreans [30]. Seo et al. hypothesized that, in Korean society [30], which views parents and children as the same object, individuals experience intense loneliness due to ignoring their own feelings in order to fulfill others’ values and hopes. For instance, participants reported, “It feels futile when I ignore my own need to consider others” (59), and “It feels bitter when I cannot express my own hardship due to a desire to support the other person” (9). These statements illustrate how ignoring personal needs and hardships to maintain relational harmony contributes to a culturally unique form of loneliness. These findings are significant as they confirm the cultural uniqueness of loneliness among Koreans, as hypothesized in previous studies. This study’s findings also demonstrate that people can become lonely even if their relational needs (e.g., intimacy, a sense of bonding) are satisfied in a close relationship, confirming a different view from the general attributes of loneliness.

Emotions reveal the relationships between the individual and others, the individual and the self, and the individual and the world [75]. Clusters 1 and 2 represented the relationships between self and others, whereas Cluster 3 represented the relationship between the individual and the world (e.g., “In a rapidly changing world, it feels like I am standing still alone; (50)”, and “It is bewildering not knowing where I am heading in the face of an uncertain future (45)”). Participants reported that, when their sense of self-worth was not reaffirmed by various “achievements”, social “appraisals”, or “reactions” from others on social media, they felt disconnected from the world and left behind, causing anxiety or helplessness as they viewed themselves as “failures” or “dropouts”. Though studies have defined loneliness based on deficiencies in relational intimacy and care [19], we found that the Korean adult participants experienced loneliness when their need to realize value in their relationships with the world, beyond other relationships, was not satisfied. “Benign envy”, an emotion unique to Koreans, comes from comparing oneself to others or social norms. Benign envy enables individuals to connect with the subject of their envy and is an unpleasant and painful emotion causing isolation and avoidance at the intrapersonal level [86]. Feeling lonely from seeing others being happy on social media is consistent with the uniqueness prominent in Korean culture, which can cause people to feel left out, isolated, and empty.

Particularly, these experiences seem to be related to the social context of Korea, where high social media usage is prevalent. For instance, participants reported feeling more alone when seeing the lives of others through social media. This phenomenon was not reported among the elderly but was primarily observed in individuals in their 20s and 30s. This aligns with previous studies indicating that excessive social media use among adolescents and young adults exacerbates feelings of loneliness [87,88]. Despite the ability of social media to expand the social networks and feelings of relational fulfillment of its users, the glamorous lives that it portrays often lead its users to develop feelings of emptiness and inadequacy.

Loneliness experienced in the relationship between the self and the world is also linked to being achievement-oriented in order to prove extrinsic worth [86]. As people become more immersed in objective achievement, they are more likely to use others and themselves as tools for such achievement. East Asian collectivist cultures generally emphasize social ties and affiliations, leading to a relationally oriented and contextually sensitive self [89]. Ironically, relational sensitivity reinforces immersion in external norms, sensitivity to social comparison, and instrumental cognition to affirm the value of the relational self. In turn, this creates a structure that is sensitive to one’s psychosocial position in relationships and contexts; that is, overly achievement-oriented and competitive social structures amplify loneliness. According to the Mind sponge Theory [37], relational sensitivity strongly absorbs social comparison sensitivity. As a result, even individuals whose relational needs are fulfilled may experience amplified feelings of emptiness and loneliness if they maintain a poor self-representation in terms of relational status or influence. 

The second sub-cluster of Cluster 3, “Bewilderment like a boat adrift in a vast sea”, refers to the lonely frustration felt in an uncertain and fast-paced life. Loneliness is experienced within the relationship between self and the world. This is similar to existential loneliness in that it represents emptiness from the fundamental sense of separation, unlike helplessness from being excluded due to social standards [90,91]. As humans experience an existential vacuum when they lose the motivation to seek meaning [74], this study’s findings suggest that individuals may experience loneliness when they are unable to discover their identity and find meaning in life. Existential perspectives on loneliness have been studied mostly through phenomenological methods but are not included in measuring loneliness [30]. However, this study’s findings demonstrated that discouragement in seeking meaning and non-existent relationships are critical elements of the loneliness experienced by participants.

### 4.3. Relevance of Elements of Loneliness 

Among the 80 statements derived in this study, the participants rated “There is no one I can deeply share my thoughts and feelings with”, “I do not have anyone to lean on or rely on (14)”, “I feel worthless when my worth is not acknowledged (29)”, and “I feel like I have to deal with my emptiness or difficult emotions on my own (62)” as the most relevant. The actual situation or perception of lacking someone to share important parts of life with, rely on, and reciprocally comfort were reconfirmed as key elements of loneliness experienced by Korean adults. Moreover, proving one’s self-worth, which was not considered a fundamental element of loneliness in previous studies, was recognized as significant to this study’s participants. 

When relevance was examined by cluster, the participants perceived “Emotional distance with oneself or with others in a relationship”, “Powerlessness and emptiness due to being directionless”, and “Emotional distress due to actual or anticipated absence of connection in relationships” to be the most relevant elements of loneliness. Among the sub-clusters, “Dissatisfaction due to unmet expectations in a close relationship” and “Powerlessness felt during difficulties” were the most relevant. The findings revealed that feeling powerlessness in proving one’s self-worth during difficulties and solitude during periods of uncertainty were perceived to be more relevant as elements of loneliness than the lack of a relationship, highlighting aspects of loneliness that previous research has examined. 

Koreans consider positive self-perception and effectively controlling their lives as elements that influence quality of life and psychological well-being [92,93]. Hopelessness about the future due to the low likelihood of achieving one’s desired goals despite various efforts is also a key element that diminishes happiness among Koreans [94,95]. Koreans’ tendency to establish their happiness and goals based on overcoming future uncertainties and the instability of social status, employment, and achievements worsened during the COVID-19 pandemic [95]. Though having someone close is important, participants reported that firmly establishing their own values by overcoming instability would alleviate their inner suffering and loneliness. These statements reflect the desperation of people living in increasingly competitive and uncertain times.

Further, there were differences in the perceived relevance of the elements of loneliness between the subgroups. Women perceived “Fear of being alone” to be more relevant than men, whereas men perceived “Bewilderment like a boat adrift in a vast sea” to be more relevant than women. These findings were consistent with gender difference studies reporting that women are more relationship-oriented and elevate connection with others more than men, whereas men emphasize independence and personal achievements more than women [96,97,98]. Results regarding women and men who feel lonelier when alone during times of uncertainty should be reconfirmed, and gender differences should be considered when developing interventions for lonely individuals. 

Regarding age-based differences, participants aged 20–49 years perceived “Dissatisfaction due to unmet expectations in a close relationship” and “Solitude due to the absence of intimate relationships” as the key loneliness sub-clusters, unlike those aged ≥50 years. The lack of people to share emotional and social bonds with was perceived as the loneliest experience by those aged 30–39 years, whereas “Bewilderment like a boat adrift in a vast sea” and “Powerlessness felt during difficulties” were perceived as critical by those aged ≥50 years. Older participants who perceived themselves as being relatively closer to death considered existential loneliness as relevant, suggesting that they have painful experiences due to frustrations of proving their social worth. The lack of social contact and isolation among older adults is a serious issue [99,100], suggesting that problems associated with self-worth and self-efficacy are major risk factors for the mental health of this population. More research is needed to foster support and interventions to address this issue. 

With respect to differences by occupation, the sub-cluster “Solitude due to the absence of intimate relationships” was perceived as the most relevant among college students but the fourth most critical among homemakers, office workers, and older adults. Compared with the older population, young people highly associate loneliness with the presence or absence of physical time spent in actual relationships [62]. Thus, although some positive relationships are important, the perception of a negative situation of feeling isolated with no friends can cause even greater loneliness [62]. Similarly, relatively younger college students perceived a lack of relationships as critical. Conversely, office workers rated the sub-cluster “Bewilderment like a boat adrift in a vast sea” to be more relevant than other groups, indicating that they perceived emptiness due to ignoring important things in a fast-paced world as meaningful. By contrast, “Fear of being alone” was perceived as the least important among homemakers and older adults since the fear of being alone due to disconnection from social relationships was a sub-cluster of loneliness that was acceptable to a certain degree.

### 4.4. Implications for Practice, Advocacy, Education and Training, and Research

Loneliness is a complex and universal emotional experience that severely affects individuals [75]. Nevertheless, few studies have examined the cultural differences in loneliness (e.g., [42,57,101]). By empirically exploring how Korean adults conceptualize loneliness, this study presents several implications. First, through a comprehensive review of statements, dimensions, and clusters, which were consistent with fundamental characteristics established in previous studies, the loneliness experienced by Korean adults was defined as a “painful and sad emotion from a loss of or damage of self-worth influenced by negative perception of oneself or being disconnected from relationships with self, others, and the world” [30]. The fundamental elements of loneliness, such as unmet relational needs, negative criticism, and painful emotions, emphasized in previous studies emerged in this study, suggesting that Koreans also experience culturally universal characteristics of loneliness. The statements derived in this study included the contents of all 20 items of the UCLA Loneliness Scale. Three items in the UCLA Loneliness Scale on extraversion and shyness were not included in this study’s statements because they entail personality traits related to loneliness, not the fundamental aspects of loneliness. 

Second, we identified major aspects of loneliness that should be examined beyond existing conceptual discussions. This study’s findings suggested that “unmet relational needs”, a key concept of loneliness, should be specified in a multidimensional manner. Previously, relational needs were recognized as a target-oriented concept that can be fulfilled by others. However, this study’s findings revealed that “self-focus” is a key axis in the conceptual structure of loneliness. In addition to the “relational needs” emphasized in previous studies, participants experienced loneliness due to frustrations related to self-actualization, existential needs, or needs for individuality. Accordingly, the findings suggest that clients who have anxiety and fear of falling behind despite their efforts to overcome academic or employment challenges are likely to experience loneliness in their struggles to prove their worth. The findings also indicate that, even if the sense of connectedness with others is satisfied, some clients may experience fusional loneliness from a loss of self and individuality due to extreme closeness.

We submit that the existing approach, which limits the source of loneliness to relationships and presents loneliness only as frustration associated with relational needs, may diminish the concept of loneliness and the scope of loneliness experienced by Korean adults. If counselors rely on this narrow definition of loneliness, they are likely to fail to link their clients’ emotional distress with loneliness. It has been found that frequent feelings of loneliness are not recognized in many cases [102]. When professionals have an expanded understanding of loneliness and recognize it in their clients, they can apply appropriate interventions. From the client’s perspective, being able to clearly recognize ambiguous and subconscious emotions enables them to understand how they are evaluating their own needs, hopes, and goals, which can be transformed into a new, more adaptive meaning [103]. This study’s findings can spur multidimensional discussions on loneliness.

Third, studies have classified loneliness into depression, sadness, discouragement, and sorrow, and, further, into sadness or sadness–depression groups [65]. These emotions share the characteristics of inactivity as an emotional response to loss [36]. This can be viewed as being close to the emotional classification associated with “emotional lack”, similar to that in this study. However, considering “self-focus” in the “focus” dimension, loneliness can be classified as a self-conscious emotion. The sources of self-conscious emotions are self-criticism and criticism of relations with others [65,104], which relate to shame, guilt, embarrassment, and jealousy. Self-focus reflects emotions felt in the relationship with the self, whereas other-focus includes the motivation to satisfy one’s own needs. Lonely people exhibit self-consciousness in social situations [105]. 

Loneliness may, therefore, be viewed as a category of self-conscious emotion. If loneliness is induced due to the influence of self-assessment and perceptions of oneself within a relationship, then it has an important social function similar to self-conscious emotions, indicating that the cultural context must be considered to understand loneliness [105]. The self may be perceived differently depending on the culture, leading to different conceptualizations of self-conscious emotions. For instance, many North Americans feel strong self-conscious emotions from assessing their own actions, whereas self-conscious emotions are triggered in Asians by strongly reflecting the activities or actions of family members on their own individuality [106]. Considering the characteristics of the self-conscious emotions of loneliness, comprehensive studies on cultural differences in loneliness are needed. In addition, self-consciousness may be chronic, even when the situation that elicited it has passed, which can lead to its internalization as a personality trait [107]. For instance, when shame is experienced for an excessively long time, it becomes internalized as a self-identity and deep feelings of inferiority and inadequacy [107]. An inward focus can promote deeper self-awareness, leading to a positive effect on emotional regulation. Nonetheless, excessive inward focus can exacerbate pathological symptoms such as depression and anxiety [108,109,110,111]. A meaningful understanding of the complex and multidimensional process of loneliness, including chronic loneliness, and the factors or situational characteristics that cause loneliness is required. 

Fourth, “Powerlessness felt during struggles” and “Bewilderment like a boat adrift in a vast sea” include emptiness [111], reflecting existential loneliness. Existential loneliness and its philosophical approach have been discussed theoretically [112], with loneliness conceptualized from an extreme philosophical or unscientific viewpoint due to the lack of valid and reliable data [113,114]. In a meta-analysis of 143 qualitative studies on loneliness, less than 20 conceptualized existential loneliness [21]. However, the present study found that existential loneliness is critical among Korean adults, presenting implications for loneliness interventions. Improving social skills, strengthening social support, expanding opportunities for interactions, and addressing maladaptive social cognition have been proposed as effective interventions for loneliness [19,46,115]. However, the present findings suggested that providing opportunities to reflect on the meaning of one’s own existence and firmly establish one’s identity in life could be highly effective in alleviating loneliness. For individuals who feel emptiness from alienating themselves while trying to meet social demands, the process of finding an integrated new self may be more crucial than improving and expanding social relationships. Researchers have proposed strategies for transforming periods of loneliness into periods of solitude for lonely individuals by repositioning time spent alone as time for connecting with and caring for oneself [30,116]. To build the strength to be alone, professionals should help lonely individuals confront their existential loneliness and experience the relational change with their alienated inner world.

Fifth, loneliness has been classified as emotional and social loneliness based on Weiss’s binary classification [26]. However, our findings showed that this binary system may be an ambiguous and undifferentiated classification for Korean adults. The loneliness identified in this study can be divided into four types: (1) the lack of a relational target in one’s psychological space; (2) the existence of a target, but no sense of connectedness; (3) the existence of a target, but with the loss of self due to being too close; and (4) the loss of self within one’s own psychological space. The findings indicate the need for four different types of power in situations where loneliness is experienced: (1) the power to face and tolerate emptiness in one’s psychological space; (2) the power to build a connection with the target who has entered the psychological space; (3) the power to recognize the need to keep an appropriate distance in a relationship and establishing boundaries; and (4) the power to consider the self in one’s psychological space as autonomous and meaningful. It may be that, when Korean adults are unable to hold such power, they become tired and lonely. 

Sixth, this study is significant in that it explored the loneliness experienced by Korean adults and extended the discussions on loneliness by Western studies. We collected the experiences of participants in different developmental life stages, and identified differences in their perceptions, thereby reconfirming the contextual relevance of loneliness. Understanding differences in loneliness by types and groups while considering the sociocultural context can present meaningful information for clinical practice. The findings provide foundational data for developing scales to measure loneliness that account for the sociocultural context, based on a comprehensive understanding of the contents and components of loneliness. 

### 4.5. Limitations and Recommendations for Future Research

This study has several limitations. First, we included various relational, cognitive, and emotional elements in the derived statements; however, relatively few physical symptoms were identified. To understand subjective emotional experiences such as loneliness, the definition should comprise emotions, thoughts, and behaviors since emotions are accompanied by behavioral tendencies [33,103,117]. Individual participants mentioned behavioral elements such as “crying”, “becoming dazed”, and “energy drained from the body”. However, statements on behavioral responses in the overall loneliness context were not included as contents applicable to specific statements. Although the participants mentioned highly individual characteristics, only statements that were deemed generalizable and could be rated by others were included. Accordingly, in the future, behavioral responses to loneliness should be specified and considered in conceptualizing it.

Second, this study compared the mean relevance among subgroups based on gender, age, and occupational characteristics to examine patterns in the differences in the relative relevance of loneliness among groups. Though the findings showed statistically significant differences in relevance among some clusters, some results without statistically significant differences may have been considered due to the nature of the pattern-matching analysis. Concept mapping is insufficient for statistically testing differences between individuals or subgroups [118]. Moreover, the results should be interpreted carefully as some groups (e.g., older adults) were very small. To effectively test differences among groups, a multidimensional scale that reflects the types of loneliness experienced by Korean adults should be developed. 

Third, although the study was primarily conducted in 2022, during the COVID-19 pandemic, the research design included measures to control for the pandemic’s impact. As such, the results focus on chronic elements of loneliness rather than temporary changes due to the pandemic. However, it is worth noting that the study participants might have experienced different aspects of loneliness due to the pandemic, which this study did not specifically address. Future research should explore how COVID-19 has impacted loneliness and whether the elements and characteristics of loneliness have changed post-pandemic. This could help identify necessary coping strategies for potential future pandemics. Additionally, while our conclusions are based on cultural characteristics observed in prior studies on Korea, it has not been confirmed that these features are unique to Korea. Further research is needed to determine whether these characteristics are indeed distinctive to Eastern cultures in general and to Korea in particular, as comparative studies in Western contexts are limited.

Fourth, interviews were conducted with 47 participants; however, only 26 participants, excluding 6 new participants, participated in the statement classification and rating. In particular, many older adults had difficulty using a computer and struggled with the complexity of the tasks; consequently, they were unable to participate in the classification and rating despite their willingness. We created statement cards with larger fonts and physically met with some participants for the classification process. Nevertheless, we were unable to meet with more participants due to the COVID-19 pandemic, whereas others discontinued their participation after testing positive for COVID-19. Methods that allow older adults to participate in the classification and rating of longer, more complex concept mapping are required. 

Finally, data were collected using a qualitative research method; thus, the samples were determined by conceptual requirements, not representativeness [119]. Unlike quantitative research, which prioritize larger sample sizes to reduce standard errors and enhance population estimates, qualitative research operates on the philosophy that a smaller sample size is sufficient if it allows for a thorough understanding of the research subject [120]. Although this study was meaningful in that it collected empirical data, the demographic characteristics were limited since there were only 47 participants. In particular, the older adult population had significantly lower Internet use than other groups; possibly, only Internet-savvy older adults participated in the Internet-based recruitment and data-rating processes. Caution should, thus, be taken in generalizing the findings. Larger sample sizes are necessary in future research to confirm whether this study’s findings can be replicated in various age groups.

## 5. Conclusions

Loneliness is an incredibly powerful and complex emotion [22]. Therefore, managing loneliness is crucial for enhancing mental health and quality of life [112]. It is, thus, imperative that mental health professionals pay greater attention to this issue [82]. To address the emotional and social needs of various populations through effective interventions, it is important to consider the cultural and contextual factors of loneliness. Our findings include not only the fundamental elements of loneliness revealed in previous studies, such as the lack and deprivation of relational social connections, but also the intense expectations of close relationships (interdependence), collective identity, lack of relational self-assurance, and loss of self in overly close relationships—features not previously highlighted. We hope that this study provides a foundational basis for accurately measuring loneliness and developing effective approaches to alleviate it across diverse cultural setting.

## Data Availability

The data presented in this study are available upon request from the corresponding author.

## Appendix A

**Table A1:** Statements on the loneliness experienced by Korean adults.

Cluster	Statement	Relevance *M* (*SD*)
Cluster 1	Emotional distress due to actual or anticipated absence of connection in relationships	3.64 (0.97)
Sub-cluster 1	Solitude due to the absence of intimate relationships	3.73 (0.96)
12	There is no one I can deeply share my thoughts and feelings with.	4.44 (0.80)
14	I do not have anyone to lean on or rely on.	4.38 (0.87)
13	When difficult times arise, there is no one to help me.	4.16 (0.88)
11	I feel lonely because I want comfort but cannot express my feelings.	4.16 (0.63)
52	There is a void due to the death of parents, family members, etc.	3.97 (1.03)
44	In the organization I belong to, there is no one to share my feelings with.	3.88 (0.75)
80	When I am alone on important days (e.g., birthdays), I feel like I am inadequate.	3.88 (1.07)
16	I feel empty when I am away from loved ones.	3.84 (0.88)
41	When I cannot relate to or participate in the topic of conversation at gatherings, I feel like I am the odd one out.	3.81 (0.90)
61	There is no one who values me as much as I value myself.	3.56 (1.05)
77	There are not many people to share my interests and hobbies with.	3.50 (0.92)
38	I feel lonely because I cannot engage in physical intimacy with my loved one.	3.41 (1.13)
17	There is limited interaction or contact with people within the organization.	3.31 (1.03)
Sub-cluster 2	Fear of being alone	3.51 (0.98)
62	I feel like I have to deal with my emptiness or difficult emotions on my own.	4.28 (0.77)
51	It feels empty because it seems like there is no one in this world to trust.	3.81 (0.86)
37	Even though I have been living diligently, I suddenly feel powerless and alone.	3.75 (0.95)
48	It feels like a precious presence will disappear from my side.	3.72 (1.05)
43	There is no one at work who supports me.	3.72 (0.92)
46	It feels like I cannot belong or establish roots anywhere.	3.69 (0.78)
69	It feels like I will not have a family and will be alone.	3.38 (1.21)
Cluster 2	Emotional distance from oneself or from others in a relationship	3.74 (0.91)
Sub-cluster 1	Dissatisfaction in a relationship due to unmet expectations	3.78 (0.91)
15	It feels like I have no one on my side if those close to me do not show interest or care about important matters.	4.25 (0.67)
2	When close relationships, such as family or lovers, become distant, it feels like I am alone in this world.	4.25 (0.80)
7	If people I thought were close to me do not fully understand me, it feels like they are distant.	4.09 (0.86)
25	It is disappointing when the other person does not care about me as much as I care about them.	4.06 (0.84)
78	Someone who was once very close (e.g., children, a very close friend) is gradually becoming distant.	4.00 (1.05)
33	When those close to me do not believe what I say, I feel like I’m disconnected from everyone.	3.88 (0.83)
26	It feels like the other person does not give their heart to me as much as I give mine to them.	3.84 (0.85)
71	When the other person does not focus on me, it feels like I am not valuable.	3.84 (1.02)
5	It feels empty when we stop having heart-to-heart conversations in a familiar relationship.	3.75 (1.11)
63	I distance myself because I fear getting hurt more if l rely on others.	3.72 (1.02)
67	When I am going through a tough time and the other person cannot fully understand or help me, it feels lonely.	3.59 (0.84)
8	Even if I express my feelings honestly, it feels like the other person will not understand.	3.53 (0.95)
68	It feels lonely when loved ones feel lonely or hurt because of me.	3.53 (1.95)
Sub-cluster 2	Self-alienation and loss of self in relationships	3.52 (0.88)
58	It is disheartening when I feel like my true thoughts and emotions are not respected.	4.19 (0.90)
72	Over time, the expectations of people and the yearning for relationships decrease.	3.72 (0.81)
59	It feels futile when I ignore my own needs to consider others.	3.44 (0.91)
9	I feel bitter when I cannot express my own hardships due to a desire to support the other person.	3.53 (0.92)
66	When loved ones are going through a tough time and I can only watch because I can’t help them, it creates distance between people.	3.22 (0.79)
57	In close relationships (family, lovers, etc.), when everything has to be shared, it feels like I am disappearing.	3.03 (0.92)
Cluster 3	Powerlessness and emptiness due to being directionless	3.65 (0.99)
Sub-cluster 1	Powerlessness felt during difficulties	3.78 (0.99)
29	I feel worthless when my worth is not acknowledged.	4.38 (0.79)
74	When I have not achieved anything, I feel worthless.	4.25 (0.92)
1	When I feel like I have not done well in relationships (roles or functions), my heart sinks.	4.03 (0.82)
30	It feels desolate because it seems like everyone else is happier than me.	3.94 (0.98)
32	Others seem to have much more than me.	3.91 (1.03)
28	When it feels like my peers are receiving more recognition, I feel left behind.	3.84 (1.05)
55	When I receive negative evaluations, I feel like a failure.	3.81 (0.82)
39	When things do not go as planned in my relationships or life, it feels like my life is not my own.	3.81 (0.86)
75	I feel like my valuable actions or things are taken lightly or not respected.	3.75 (0.88)
54	It seems like neither society nor my family needs me.	3.75 (1.02)
53	I feel like a useless person in this society.	3.63 (0.94)
73	Sometimes, not only others, but I also do not believe in myself.	3.63 (1.01)
34	When I cannot fit into the mainstream, I feel useless.	3.56 (0.95)
50	In a rapidly changing world, it feels like I am standing still alone.	3.56 (1.08)
31	When I see happy people on social media, it feels like I am the only one falling behind.	3.53 (0.95)
Sub-cluster 2	Bewilderment like a boat adrift in a vast sea	3.63 (1.04)
45	It is bewildering not knowing where I am heading in the face of an uncertain future.	4.06 (0.98)
21	Being too busy, I miss out on precious and important things.	3.88 (1.04)
22	I am so busy that I do not even have time to focus on my own thoughts and feelings.	3.81 (1.09)
60	When I look back on my life, it feels like I can never go back to those times.	3.59 (1.13)
47	The idea of someday dying and disappearing from this world feels empty.	3.19 (1.00)
